# Counterclockwise block-by-block knowledge distillation for neural network compression

**DOI:** 10.1038/s41598-025-91152-3

**Published:** 2025-04-03

**Authors:** Xiaowei Lan, Yalin Zeng, Xiaoxia Wei, Tian Zhang, Yiwen Wang, Chao Huang, Weikai He

**Affiliations:** 1 School of Information Science and Electrical Engineering, Shandong Jiaotong University, Jinan, 250357 China; 2 School of Information Engineering, Shandong Management University, Jinan, 250357 China; 3 Engineering Training Center, Shandong University, Jinan, 250100 China; 4 Jiefangqiao Fire Rescue Station, Lixia District Fire Rescue Brigade, Jinan, 250014 China

**Keywords:** Deep neural networks, Model compression, Knowledge distillation, Progressive blockwise knowledge distillation, Software, Information technology

## Abstract

Model compression is a technique for transforming large neural network models into smaller ones. Knowledge distillation (KD) is a crucial model compression technique that involves transferring knowledge from a large teacher model to a lightweight student model. Existing knowledge distillation methods typically facilitate the knowledge transfer from teacher to student models in one or two stages. This paper introduces a novel approach called counterclockwise block-wise knowledge distillation (CBKD) to optimize the knowledge distillation process. The core idea of CBKD aims to mitigate the generation gap between teacher and student models, facilitating the transmission of intermediate-layer knowledge from the teacher model. It divides both teacher and student models into multiple sub-network blocks, and in each stage of knowledge distillation, only the knowledge from one teacher sub-block is transferred to the corresponding position of a student sub-block. Additionally, in the CBKD process, deeper teacher sub-network blocks are assigned higher compression rates. Extensive experiments on tiny-imagenet-200 and CIFAR-10 demonstrate that the proposed CBKD method can enhance the distillation performance of various mainstream knowledge distillation approaches.

## Introduction

Deep learning stands as a pivotal direction in the realm of artificial intelligence^1^, revolutionizing the field and successfully driving the progress of various real-world applications, such as image classification^2,3^, object detection^4,5^, text classification^6,7^, and machine translation^8,9^. The powerful capabilities of deep learning models often stem from their vast number of parameters. However, most models are too computationally expensive to run on mobile or embedded devices. Therefore, in many industrial sectors, there is a demand for lightweight deep learning models, as these smaller models are suitable for deployment on end-user devices. Consequently, the current research focus lies in effectively reducing the size and computational requirements of deep learning models without compromising their performance. Currently, there are five primary strategies to achieve efficient and lightweight neural network models: the direct design of lightweight neural network models^10–12^, pruning^13^, quantization^14,15^, network automation design leveraging neural architecture search^16,17^, and knowledge distillation.

Due to its ability to effectively address the contradiction between the complexity of deep learning models and limited computational resources, as well as its strong model generalization capability, knowledge distillation has garnered significant attention from researchers in recent years. Knowledge distillation aims to transfer knowledge from a robust and large model (known as the teacher model) to a more lightweight model (known as the student model). The concept of knowledge distillation is attributed to the contributions made by Hinton in their groundbreaking work^18^. The proposed knowledge distillation method transfers knowledge from the teacher model to the student model by making the output of the student model closer to the soft target outputs generated by the teacher model. However, the significant capacity difference between the teacher and student models results in a “generation gap.” To address this issue, the research by Gotmare et al. theoretically demonstrates that complex teacher models and simple student models exhibit significant capacity differences in their intermediate hidden layers, leading to different feature representation abilities^19^. Therefore, merely acquiring the teacher’s output feature knowledge is insufficient. By transferring the intermediate layer knowledge of the teacher model, it helps to bridge this gap. Based on the above analysis, various novel knowledge distillation techniques have been proposed, including Progressive Block-wise Knowledge Distillation (PBKD) by Wang^20^. PBKD gradually replaces teacher subnet blocks with corresponding student subnet blocks, proceeding from shallow to deep layers. During each stage of the block replacement process, the other subnet blocks within the teacher network remain unchanged, enabling the student model to acquire intermediate layer feature knowledge from the teacher model. At each stage, the structural similarity between the teacher and student models narrows the generational gap. Furthermore, PBKD introduces a set of design principles for student subnet blocks, stipulating that the channel dimensions of student subnet blocks should be reduced from their corresponding teacher subnet blocks to ensure that both subnet blocks have the same receptive field and depth. Additionally, Blakeney et al. proposed a parallel block-level distillation method that identifies all compressible layers in the teacher model and creates individual tasks for their replacement^21^. Once all layers are successfully replaced, the main MPI process aggregates the weights from all processes and combines them into a new compressed model. Although this method further reduces training time, it primarily relies on the intermediate layer losses of the teacher and student subnet blocks, and therefore cannot transfer knowledge related to high-level semantic features such as logistic regression values. Meanwhile, both of the aforementioned block-level knowledge distillation methods apply the same channel reduction ratio to all subnet blocks of the teacher model, ignoring the inherent characteristic differences between subnet blocks at different locations, which may lead to a decrease in the overall accuracy or efficiency of the compressed model.

Dropout is a regularization technique designed to prevent overfitting in deep neural networks by randomly shutting down a portion of neurons during the training process, thereby enhancing the model’s generalization ability^22^. This concept provides inspiration for our research. In each stage of Progressive Blockwise Knowledge Distillation (PBKD), an attempt is made to replace the teacher subnet block with a student subnet block from a subnetwork. However, due to the generation gap between the teacher and the student, each replacement process incurs information loss, similar to the impact of randomly discarding subnodes. Neural networks that utilize dropout typically employ a lower dropout rate at shallow layers to avoid losing too much input data, while using a higher dropout rate at deeper layers. This prompts us to consider whether a similar approach can be adopted in PBKD.

In summary, this paper proposes a counterclockwise block-wise knowledge distillation method for neural network compression, with the main contributions outlined as follows:

1. Based on Progressive Blockwise Knowledge Distillation, an innovative method for adjusting the compression rate according to the depth of the network is proposed, enabling a higher channel reduction ratio to be achieved in deeper subnet blocks. This strategy not only enhances distillation efficiency but also allows the generated academic subnet blocks to achieve higher classification accuracy with similar computational resource consumption.
2. By introducing the concept of compressed block-wise distillation, a novel multi-stage knowledge distillation method named CBKD (Compressed Blockwise Knowledge Distillation) has been developed. This method can more effectively utilize the knowledge from the teacher network and transfer it to the student network, demonstrating superior distillation performance among various mainstream knowledge distillation techniques.

The remainder of this paper is organized as follows. Section 2 introduces the concept of knowledge distillation and the existing problems. Section 3 presents the design process of a new knowledge distillation method, CBKD. Section 4 provides the experimental results of the proposed method. Finally, Section 5 concludes the paper.

## Related work

*Knowledge distillation* The core concept of knowledge distillation involves transferring “knowledge” from a teacher model, which is typically a large and high-performance model, to a student model, which is typically a smaller and lightweight model. This transfer aims to enable the student model to acquire the reasoning and generalization capabilities of the teacher model. At present, knowledge distillation can be divided into four main types based on the type of knowledge transferred: (i) output feature knowledge^23^: output feature knowledge usually refers to the final layer features of the teacher model, mainly including logical unit knowledge and soft target knowledge. The basic idea of output feature knowledge distillation is to enable the student model to learn the final prediction of the teacher model, thereby achieving the same prediction ability as the teacher model. Although the original knowledge distillation was proposed for classification tasks and only included inter-class similarity as soft target knowledge, in other tasks such as object detection, the final layer feature output of the network may also contain information related to object localization. (ii) Transfer intermediate feature knowledge^24,25^: This method focuses on extracting features from the network layer of the teacher model to provide guidance for the intermediate layer of the student model. (iii) Knowledge of relational features^26,27^: This approach considers that the essence of learning is not in the output of features, but in the relationships between layers and sample data. It mainly emphasizes providing consistent identity mapping, enabling the student model to better grasp the relational knowledge of the teacher model. (iv) Structural Feature Knowledge^28,29^: This strategy involves conveying the comprehensive knowledge system of the teacher model, encompassing not only the output layer knowledge, intermediate feature knowledge, and relational feature knowledge but also the spatial feature distribution and other aspects of the teacher model knowledge.

*The generation gap between teacher and student models* In the context of knowledge impartation and educational processes, disparities in understanding, experience, and language between educators or experts and students can hinder effective information transmission, leading to learning obstacles or misunderstandings. During the process of knowledge distillation, structural differences (i.e., the generation gap) between teacher and student models can result in information loss. FitNets^30^ mitigates this generation gap by aligning the outputs of the student model’s hidden layers with those of the teacher model’s hidden layers to transfer intermediate-level knowledge. Cho et al.^31^ and Mirzadeh et al.^32^ have identified several potential reasons for the mismatch in teacher and student capabilities. For instance, excessively strong teacher capabilities may impede the student model’s ability to emulate similar behaviors. Additionally, increased certainty in the teacher’s predictions leads to less “soft” logits (soft targets). To validate their hypotheses, they conducted a series of experiments and proposed solutions to address this issue. Cho et al. recommended optimizing the knowledge distillation process by strategically halting it in advance. Meanwhile, Mirzadeh et al. sought to assist students in model learning by incorporating assistant models.

## Our approach

### CBKD

To better understand our instructions, we provide a detailed explanation of the symbols used in this article in Table 1.

**Table 1:** Summary of notations.

Term	Definition
T	Teacher model
S	Student model
$L^{N}_{local}$	Loss of the between teacher Nth subnet block and student Nth subnet block
$L_{cls}$	Cross entropy loss
$\lambda$	A hyper-parameter used to balance the $L^{N}_{local}$ and $L_{cls}$
$f_{N}$	The function that maps an input image to the activation at block N of Teacher Model
$s_{N}$	The replacement block for Nth Teacher block

A teacher network T can be represented as a composite function of its k subnet blocks (as is shown in Formula 2), while a student network S as a composite function of its k student subnet blocks (Formula 1), where the circles represent the connections between different network modules.

1 $$\begin{aligned} T= & C \circ f_N \circ f_{N-1} \cdots \circ N_1 \end{aligned}$$

2 $$\begin{aligned} S= & C \circ s_N \circ s_{N-1} \cdots \circ s_1 \end{aligned}$$

**Figure 1:**
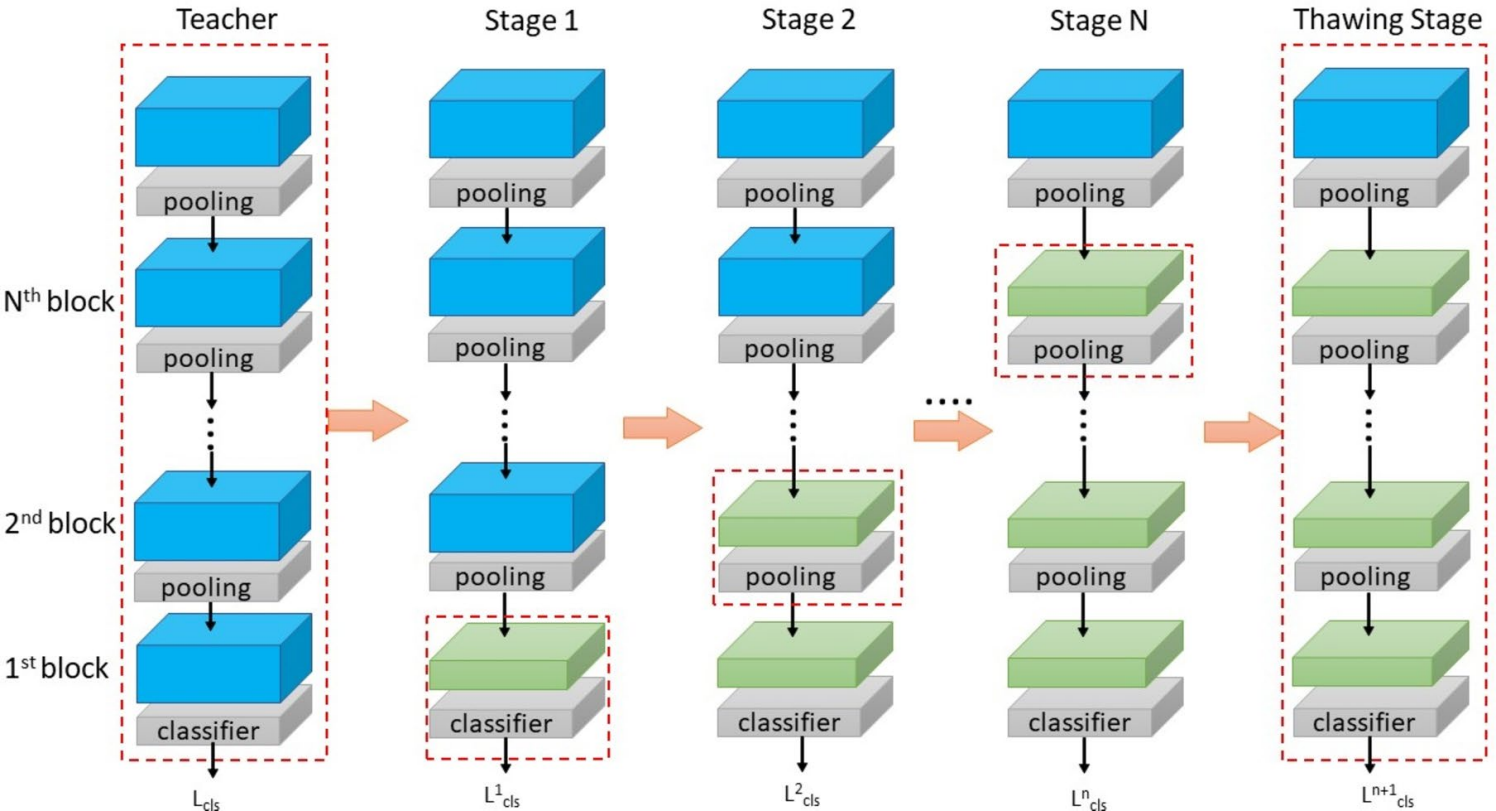
Overview of CBKD.

The comprehensive process of CBKD is vividly depicted in Fig. 1. The red dotted box indicates the subnet block trained for the current stage. Specifically, the shallowest segment of the teacher network is directly preserved within the corresponding shallow layer of the student model, reflecting a direct inheritance of foundational knowledge. The remainder of the teacher network is meticulously structured into N subnet blocks, each encapsulating unique layers of depth and complexity. CBKD is strategically segmented into N+1 phases, each playing a crucial role in the transfer of knowledge. During the initial N phases, the distillation loss serves as the guiding beacon, facilitating the transfer of knowledge from the teacher model to the student model. This transfer occurs progressively, moving from the shallowest teacher subnet blocks to the deepest, with each student subnet block mirroring its teacher counterpart in terms of knowledge acquisition. This layered approach ensures a gradual and thorough assimilation of knowledge. An intriguing aspect of CBKD lies in its phased training methodology. In the first N-1 phases, a meticulous focus is placed on the sequential training of each student subnet block. During these phases, only one student subnet block is actively trained, while the remaining networks within the student model remain in a frozen state. This isolated training environment allows for a detailed examination and refinement of each subnet block, ensuring optimal performance before integrating it into the larger student model. At the pivotal N-th phase, a transformation occurs. All parameters within the student model are unfrozen, marking the commencement of a comprehensive training session. This comprehensive training is designed to fine-tune the entire student model, reducing its reliance on the teacher model and fostering the discovery of a more suitable parameter distribution. This final phase serves as the culmination of the CBKD process, preparing the student model for independent operation with enhanced performance and reduced complexity.

Progressive Block Knowledge Distillation (PBKD) leverages a dual-pronged approach in defining its distillation loss for each stage. Specifically, it employs both the intermediate layer loss, as clearly delineated in Formula 3, and the output layer loss, comparing the outputs of the teacher and student models. This holistic consideration of losses at multiple levels ensures a comprehensive transfer of knowledge from the teacher to the student model. In stark contrast, Cross-layer Block Knowledge Distillation (CBKD) adopts a more streamlined approach. It solely focuses on the output layer loss between the student and teacher models, consciously omitting the losses calculated between corresponding student and teacher subnet blocks. This decision stems from the recognition that teacher and student subnet blocks often possess vastly different structures and capacities, making a direct comparison of their outputs impractical and potentially misleading. Instead of enforcing a rigid alignment between these subnet blocks, CBKD respects their inherent differences and focuses on aligning their final outputs. Furthermore, by adopting a single loss function, CBKD minimizes the number of hyperparameters that need to be fine-tuned during the distillation process. This simplification not only reduces the computational overhead but also streamlines the optimization process, making it more efficient and less prone to overfitting. Experimental results have consistently demonstrated the effectiveness of CBKD’s loss function scheme. By focusing on the output layer loss and respecting the differences between teacher and student subnet blocks, CBKD is able to produce a superior student model that maintains high performance while being more compact and efficient. These results validate the robustness and practicality of CBKD’s approach in knowledge distillation.

3 $$\begin{aligned} L_{\text{ local } }^N\left( I\right) =\frac{1}{n} \sum _{i=1}^n\left( T_i-S_i\right) ^2 \end{aligned}$$

where I is the input of the network (e.g., image). In this context, ’n’ represents the area of output feature maps for both teacher and student subnet blocks, with ’T’ and ’S’ denoting the value matrices of the output feature maps for teacher and student subnet blocks, respectively.

### Student subnet block design methodology

Dropout serves as a widely employed regularization technique in deep learning. It operates by stochastically “dropping out” certain neurons of a neural network during the training process, effectively setting their outputs to zero, thereby mitigating overfitting. Given the substantial presence of input feature information in shallow networks, where each layer’s features make a relatively significant contribution to the final output, it is advisable to apply a lower dropout rate in the shallow layers of a neural network.Through experimentation, we have further observed that retaining certain network layers from the bottom of the teacher network directly leads to improved knowledge distillation performance.

**Figure 2:**
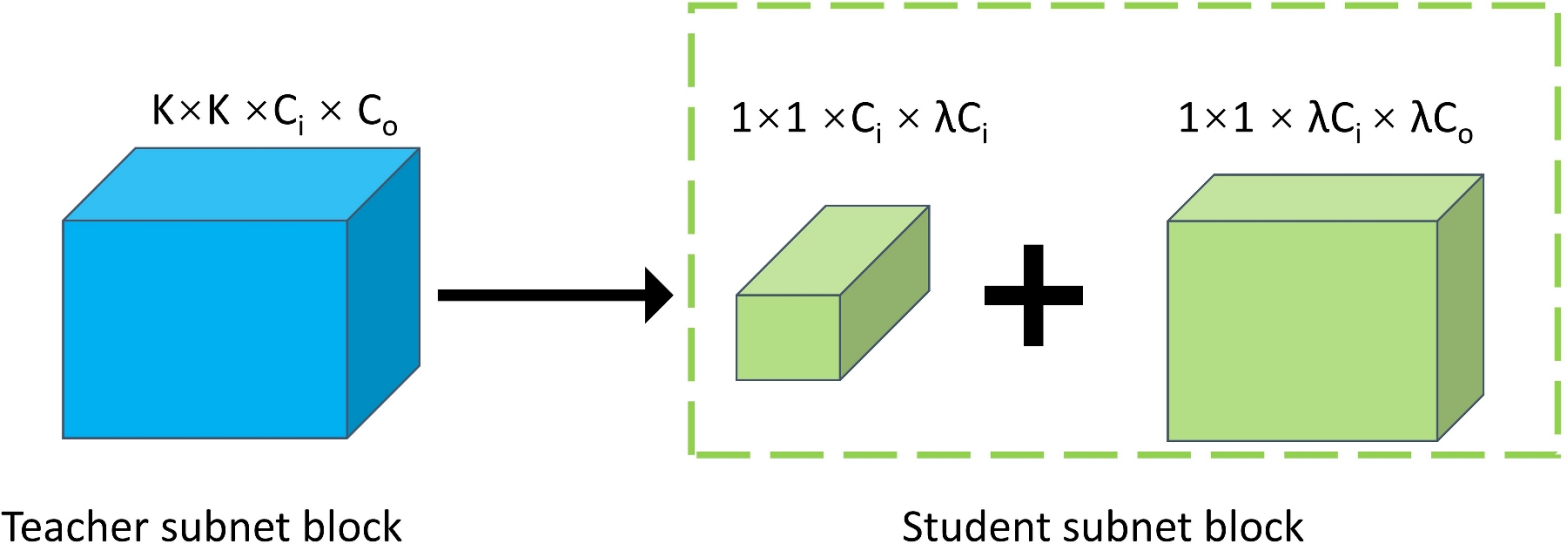
Illustration of the student subnetwork block design.

In our approach, we utilize the downsampling layer as a strategic demarcation to segment the teacher network into subnet blocks, preserving the shallowest teacher subnet block as it often contains critical information for subsequent layers. To derive the student subnet block, we systematically reduce the number of channels within the corresponding teacher subnet block. This reduction is critical for achieving model compression without compromising too much on performance. To facilitate the seamless integration of the student subnet block into the overall network architecture, we employ a 1$\times$1 convolutional layer. This layer serves a dual purpose: it adjusts the input channel number of the student subnet block to ensure compatibility with the surrounding layers, and it helps in maintaining dimensional consistency throughout the substitution process, as vividly illustrated in Fig. 2. C_i, C_o and $\lambda$ represent the input channels, output channels and compression ratio of the teacher subnet block. This design choice ensures that the student subnet block can seamlessly replace its teacher counterpart without disrupting the overall flow of information within the network. The ratio of the number of channels in the student subnet block to that in the teacher subnet block serves as a quantifiable metric of the compression applied. A lower ratio indicates a higher degree of compression, potentially leading to a more compact and efficient student model. By tuning this ratio, we can balance the trade-off between model compression and performance. Furthermore, we conducted a comparative analysis to assess the effectiveness of CBKD in different scenarios where the teacher and student subnet blocks are of varying types. This analysis was crucial for understanding the versatility and robustness of CBKD across diverse network architectures. Our findings revealed that CBKD consistently delivered impressive results, highlighting its potential as a powerful tool for model compression and knowledge distillation.

## Experiments

### Experimental details

Datasets :We ran our experiment on two datasets. CIFAR-10^33^ is a labeled subset of the 80 million small image dataset for object recognition, which contains 60,000 32 $\times$ 32RGB images in 10 categories, 5000 images per class for training and 1000 images per class for testing.Tiny-imagenet-200^34^ is a subset of Imagenet^35^, which contains 200 categories of 64 $\times$ 64RGB images, each with 500 training samples and 50 validation samples.

DNN Models :In this paper we use the widely used VGG-16^36^, Resnet18^37^ as the teacher module. It should be noted that adding a normalization layer after one or more convolutional layers is a popular practice, so we added a batch normalization layer after each convolutional layer in VGG-16.

Data Preprocessing:When experimenting on CIFAR-10 using the VGG-16 model, we simply subtract the RGB mean on the training data from each image on the dataset. For the rest of the experiments,we used the same data augmentation, that is, random cropping (crop ratio (0.6, 1.0), crop area aspect ratio (3.0/4.0, 4.0/3.0)), random horizontal and vertical flipping (probability = 0.5), normalization and random adjustment of the four attributes of brightness, contrast, saturation and hue of the image (the variation range is 0.4, 0.4, 0.4 and 0.1 in order).

Training Details:We implemented our architecture via PyTorch, we trained the network with Tesla P100 16GB GPUs on CIFAR-10 , RTX3080ti GPUs on Tiny-imagenet-200, Pytorch’s default AdamW as the optimizer, and we set up training epoches to make each distillation stage close to convergence. More training details are shown in Tables 2 and 3.

**Table 2:** Experimental details with VGG-16 as the teacher network.

Training config	VGG-16 in Tiny-imagenet-200	VGG-16 in CIFAR-10
Base learning rate	1e-3	2e-3
Weight decay	0.05	0.05
Batch size	48	100
Training epoches (CBKD)	150,200,200,200	3,8,20,20
Learning rate schedule	Cosine decay	Cosine decay
Thaw training epoches	300	20
Warmup epoches	Max((training epoches)*0.05,1)	Max((training epoches)*0.05,1)
Training epoches(Teacher)	180	30
Training epoches(Student)	300	30
Training epoches(KD)	300	30
Training epoches(FitNets)	100,300	10,20
Training epoches(RKD)	300	30
Training epoches(DKD)	300	30
Training epoches(L-S-KD)	300	30

**Table 3:** Experimental details with ResNet as the teacher network.

Training config	ResNet-18 in Tiny-imagenet-200	ResNet-18 in CIFAR-10
Base learning rate	2e-3	2e-3
Weight decay	0.05	0.05
Batch size	32	100
Training epoches(CBKD)	150,200,200,200	5,20,50,50
Learning rate schedule	Cosine decay	Cosine decay
Thaw training epoches	300	30
Warmup epoches	max((training epoches)*0.05,1)	Max((training epoches)*0.05,1)
Training epoches(Teacher)	180	30
Training epoches(Student)	300	30
Training epoches(KD)	300	30
Training epoches(FitNets)	100,300	10,30
Training epoches(RKD)	300	30
Training epoches(DKD)	300	30
Training epoches(L-S-KD)	300	30

### Ablation experiments

In this section, we have performed a total of 5 ablation experiments: (1) Comparison of loss function and optimization order selection approach; (2) Comparison of thawing training strategies; (3) Comparison of student subnet block design strategies; (4) Comparison of the number of training epoches; (5) Combined with other knowledge distillation methods. In Section 4.2.6, we compared the performance of CBKD with previous knowledge distillation methods based on PBKD.

#### Comparison of loss function and optimization order selection approach

Due to the structural differences between the teacher model and the student model, there is a generation gap in performance between them. In order to solve this problem, we hope that the student model can pay more attention to the higher-level knowledge of the teacher model, and we also hope to reduce the hyperparameters that need to be tuned by reducing the variety of loss functions, so we only select the final output of the intermediate model and the cross-entropy loss of the ground truth at each distillation stage as the loss function. Several combinations of optimization order and loss function have been compared in PBKD, and the final result is that the bottom-up optimization order and the loss function scheme of cls loss+local loss are optimal. In this section, we try two other combinations of optimization order and loss functions and compare them with the optimal scheme in PBKD. The comparison results are shown in Table 4, and the optimization order of Top-Down and the scheme of using only Lcls as the loss function are the best. Top-Down refers to distillation from the shallow layers of the teacher model to the deeper layers. Skipping-First refers to the replacement of subnet blocks in the order N-1, N-2, . . . . , 1, N.

**Table 4:** Progressive distillation performance with different loss functions and optimization orders.

Config	Optimize the order	Approach	Epoches	Accuracy change
VGG-16 (88.5%) in CIFAR-10	Bottom-Up	Lcls+Llocal(PBKD)	8,10,20,20,10	− 4.1%
	Skipping-first	Lcls (PBKD)	8,20,20,10,20	− 3.7%
	Top-down	$L_{cls}$(PBKD)	3,3,15,30,40	− 3.5%
	Bottom-up	$L_{cls}$+$L_{Local}$(CBKD)	8,20,20,10,20(thaw)	− 0.9%
	Skipping-first	$L_{cls}$(CBKD)	8,20,10,20,20(thaw)	0.6%
	Top-down	$L_{cls}$(CBKD)	3,8,20,20,20(thaw)	-**0.3%**

#### Comparison of thawing training strategies

We try to reduce the dependence of the student model on the teacher model and make it find a more suitable parameter distribution by thawing the student model obtained in the last distillation stage of CBKD. In addition, we believe that when thawing training, the learning rate setting is also a hyperparameter that should be valued, because too small a learning rate setting will make the student model unable to get out of the previous local best position, and too large a learning rate setting may make the student model forget too much of the knowledge transmitted by the teacher model. The results in Table 5 show that it is most effective to increase the thawing training stage at the end of the counterclockwise progressive distillation phase and use a learning rate close to the distillation stage. “lr” uses the same learning rate settings as the distillation stage.

**Table 5:** Results of different thawing training methods.

Freeze?	CIFAR-10		Tiny-imagenet-200	
	VGG-16 (88.5%)	Resnet-18 (85.0%)	VGG-16 (59.4%)	Resnet-18 (57.3%)
No	85.8%	82.4%	55.9%	54.2%
Yes(lr*0.1)	86.5%	83.4%	57.9%	55.0%
Yes(lr*0.25)	87.3%	84.8%	58.7%	55.2%
Yes(lr)	**88.2%**	**85.6%**	**59.4%**	**55.7%**

#### Comparison of student subnet block design strategies

**Figure 3:**
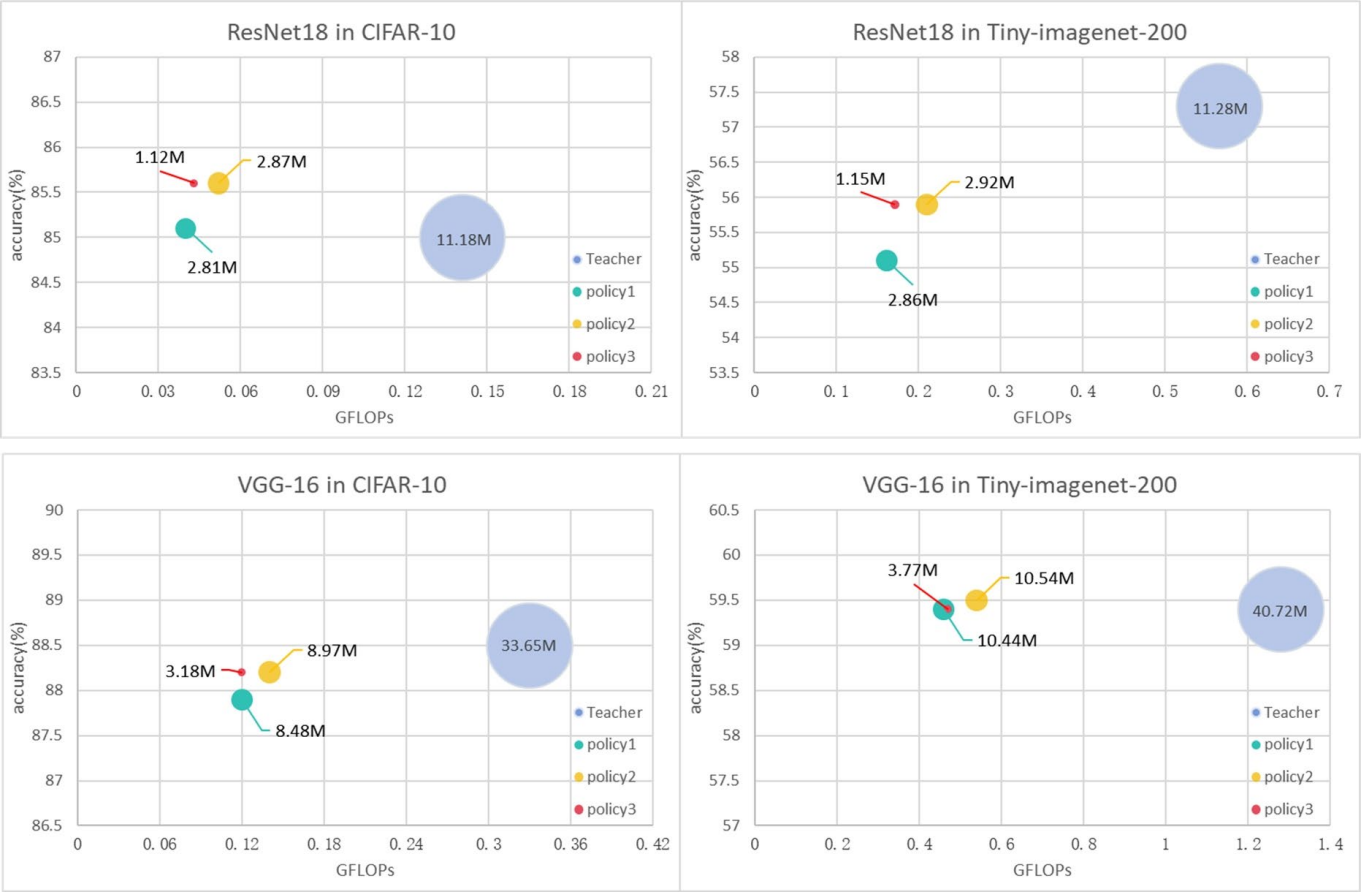
Model results with different subnet block channel reduction policies.

**Comparison of teacher subnet block compression policies**:

We applied different channel compression ratios to different subblocks of the teacher model in CBKD and compared them. As is shown in Fig. 3, reducing the compression degree of shallow subnetwork blocks of the teacher model can improve the accuracy of the student model with only a small amount of parameters and GFLOPs, but increasing the compression degree of the deep subnetwork block can not only reduce a large number of parameters, but also reduce only a small amount and even improve the accuracy of the student model, so we believe that the compression degree of the teacher subnetwork block should be increased from shallow to deep. In the figure, the width of the bubble represents the parameter size of the model. The three channel compression ratios are: Policy 1 (0.5, 0.5, 0.5, 0.5, 0.5), Policy 2 (0.75, 0.5, 0.5, 0.5), and Policy 3 (0.75, 0.5, 0.375, 0.25).

**Figure 4:**
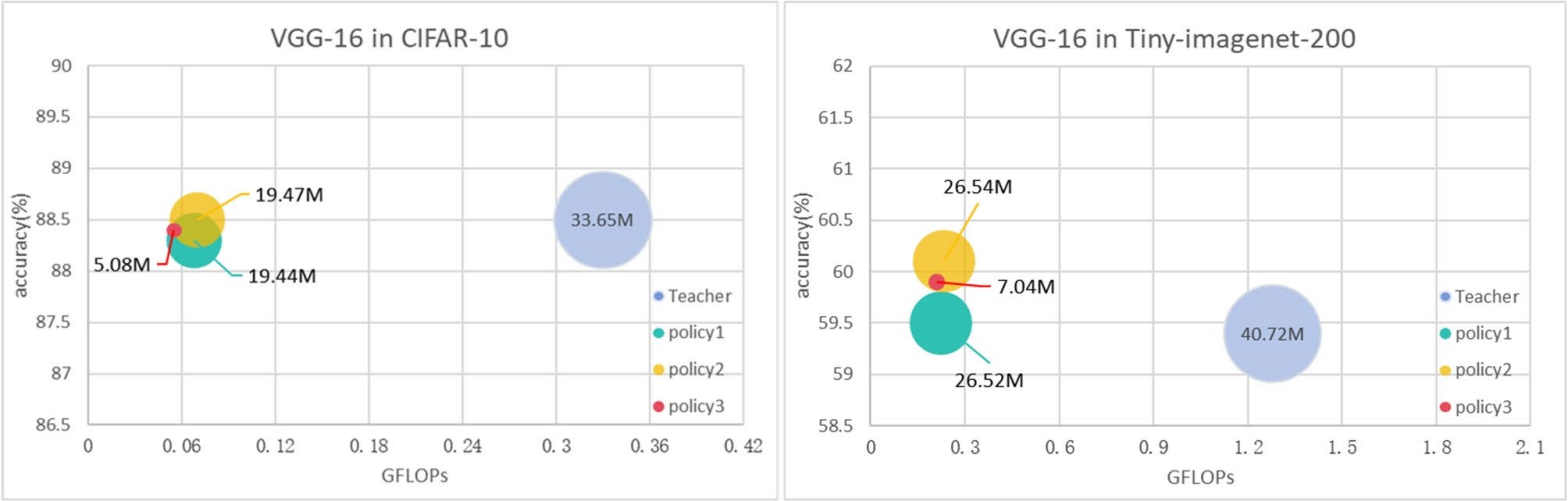
Effect of different subnetwork block channel compression strategies on heterogeneous student models.

For the student model structure that is heterogeneous with the teacher model, we also recommend increasing the compression degree of the teacher subnet block from shallow to deep. We replaced the conventional convolutional layers in the teacher model with depthwise separable convolutional layers to create the student model. This approach was employed to evaluate the knowledge distillation effects when there is a structural disparity between the teacher and student models. To support our idea, we controlled the degree of compression of the teacher subnet block by controlling the number of Depthwise Separable Convolution Layer’s channels in the heterogeneous student model, and the results in Fig. 4 confirm our opinion. In the figure, the width of the bubble represents the parameter size of the model. The three channel ratios for student subnet blocks and teacher replaceable subnet blocks are: P1 (1, 1, 1, 1), P2 (1.25, 1, 1, 1), and P3 (1.5, 1, 0.75, 0.5).

**Comparison of the number of reserved subnet blocks**:

We compared the CBKD effect of retaining the number of different teacher subnet blocks, and when the number of subnet blocks that are reserved=0, our compression ratio for the lowest subnet block of the teacher network is 0.75, and the training epoches in this stage is the same as in the previous stage. As shown in Table 6, the student model that retains the lowest subnetwork block of the teacher model tends to achieve the highest accuracy, while the student model that retains more subnetwork blocks of teachers achieves an accuracy improvement close to 0 or even negative values.

**Table 6:** Comparison of the number of reserved subnet blocks.

The number of subnet blocks that are reserved	CIFAR-10		Tiny-imagenet-200	
	VGG-16 (88.5%)	Resnet-18 (85.0%)	VGG-16 (59.4%)	Resnet-18 (57.3%)
0	87.9%	85.3%	58.8%	54.8%
1	**88.4**%	**85.6**%	**59.4**%	**56.0**%
2	88.1%	85.6%	5.9%	55.6%

#### Combined with other knowledge distillation methods

We compared the CBKD method proposed in this paper with various advanced knowledge distillation approaches. Among the compared experimental methods, FitNets^38^ belong to intermediate feature distillation, which utilizes the intermediate layers of the teacher model to guide the training of the student network. RKD^39^ falls into the category of relational feature distillation, transferring the structural relationships among multiple output samples as knowledge to the student network. DKD^40^ and logit-standardization-KD^41^ belong to logits distillation. DKD divides classification predictions into two levels: (1) binary predictions for the target class and all non-target classes, and (2) multi-class predictions for each non-target class. Based on this, DKD adjusts the influence of these two levels on the final distillation effect separately. Logit-standardization-KD standardizes the logit outputs to enhance the effectiveness of knowledge distillation. The way CBKD integrates with these methods is as follows: FitNets take the fourth subnet blocks of the teacher model as hints, while for other methods, only the loss function of each distillation stage in CBKD is modified to correspond to the integrated method.

After subjecting the student model to training using Curriculum-based Knowledge Distillation (CBKD), we proceed with further training employing KD^42^, FitNets, and RKD. The three methods belong to logits distillation, intermediate feature distillation, and relational feature distillation, respectively, and the experimental results in Table 7 show that our CBKD can effectively improve the distillation effect of these three knowledge distillation methods. KD(temperature=2, soft_loss weighted 0.2),FitNets take the fourth subnet blocks of the teacher model as hints.

**Table 7:** Effect of CBKD combined with other knowledge distillation methods.

	CIFAR-10		Tiny-imagenet-200	
Method	VGG-16 (88.5%)	Resnet-18 (85.0%)	VGG-16 (59.4%)	Resnet-18 (57.3%)
Student	87.3%	83.8%	57.6%	54.6%
CBKD	88.4%	85.6%	59.4%	55.7%
KD	87.2%	84.2%	57.6%	54.5%
KD + CBKD	88.6%	86.0%	59.6%	55.8%
FitNets	88.6%	86.6%	58.6%	54.9%
FitNets + CBKD	88.8%	86.3%	59.3%	55.6%
RWD	87.3%	84.7%	58.6%	54.9%
RWD + CBKD	88.5%	85.4%	59.3%	56.0%
DKD	87.9%	85.1%	58.2%	55.1%
DKD + CBKD	88.9%	86.6%	59.4%	55.6%
L-S-KD + KD	88.1%	85.5%	59.4%	55.5%
L-S-KD + KD + CBKD	88.7%	86.0%	59.8%	55.9%

#### Algorithm comparison

We compare the proposed method with the previous method based on PBKD, and the results are shown in Table 8, under the close FLOPS compression ratio, the student model obtained by CBKD has higher accuracy and a much smaller number of parameters.

**Table 8:** Comparison among different PBKD Methods in CIFAR-10.

Dataset	Method	Accuracy change	Param$\downarrow$	FLOPs$\downarrow$
CIFAR-10	PBKD^43^	− 3.05%	1.40$\times$	2.69$\times$
	PBKD^44^	− 0.73%	1.40$\times$	2.37$\times$
	CBKD	− **0.3%**	${\textbf {10.58}}\varvec{\times }$	${\textbf {2.75}}\varvec{\times }$

## Conlusion

In this paper,we finds that teachers should be given a greater degree of compression at the deeper network layer of the network during the Progressive Blockwise Knowledge Distillation process, and proposes a new student subnet block design criterion based on this. Compared with the previous student subnet block design of Progressive Blockwise Knowledge Distillation, it can obtain several times the reduction rate of parameter quantities and higher accuracy of student models at similar GFLOPs compression ratio. In addition, we also propose a multi-stage knowledge distillation method called Counterclockwise Progressive Blockwise Knowledge Distillation(CBKD), which can obtain a better student model and is easier to implement than the previous Progressive Blockwise Knowledge Distillation(PBKD). And it can be effectively combined with a variety of mainstream knowledge distillation methods to obtain better performance. Subsequently, we will assess the efficacy of CBKD on a broader spectrum of neural network models and datasets.

## Data Availability

The data used to support the findings of this study are available from the corresponding author upon request.
